# Drug repurposing for obsessive-compulsive disorder using deep learning-based binding affinity prediction models

**DOI:** 10.3934/Neuroscience.2024013

**Published:** 2024-06-26

**Authors:** Thomas Papikinos, Marios Krokidis, Aris Vrahatis, Panagiotis Vlamos, Themis P. Exarchos

**Affiliations:** Bioinformatics and Human Electrophysiology Laboratory, Department of Informatics, Ionian University, Corfu, Greece

**Keywords:** drug repurposing, drug repositioning, obsessive-compulsive disorder, OCD, deep learning, drug-target interaction prediction, binding affinity prediction

## Abstract

Obsessive-compulsive disorder (OCD) is a chronic psychiatric disease in which patients suffer from obsessions compelling them to engage in specific rituals as a temporary measure to alleviate stress. In this study, deep learning-based methods were used to build three models which predict the likelihood of a molecule interacting with three biological targets relevant to OCD, SERT, D2, and NMDA. Then, an ensemble model based on those models was created which underwent external validation on a large drug database using random sampling. Finally, case studies of molecules exhibiting high scores underwent bibliographic validation showcasing that good performance in the ensemble model can indicate connection with OCD pathophysiology, suggesting that it can be used to screen molecule databases for drug-repurposing purposes.

## 1. Introduction

Computer-aided drug design (CADD) refers to the use of methodologies from informatics and other adjacent fields such as cheminformatics and bioinformatics in order to design new therapeutic substances. CADD can be categorized into ligand-based and structure-based approaches [1]. Ligand-based drug design (LBDD) assumes there is knowledge about a ligand bound to a target but not for the target itself, therefore the assumption is that drugs that are similar to this ligand will also bind to that target. One method of LBDD is a QSAR (quantitative structure-activity relationship), which is the identification of patterns in the relationship between a molecule's structure and its biological activity which aids in the design of new compounds with desired properties. On the other hand, structure-based drug design (SBDD) involves designing new drugs by understanding and utilizing the three-dimensional structure of the target. SBDD assumes that designing molecules to fit the specific binding or active site of the target will lead to effective drugs. One of the most common methods of SBDD is molecular docking, a technique that samples and scores conformations of small molecules bound to a target active site.

However, de novo drug design (including CADD) involves significant time, cost, and risk commitments. It is estimated that creating a novel drug requires on average 1.24 billion dollars and 10–17 years, and entails an astonishing 45% failure rate. Drug repurposing, the process of unveiling new indications for existing drugs, can diminish those cost and time requirements by approximately 60% while greatly reducing the failure rate [2]. One successful example of ligand-based drug repurposing was observed in the works of [3], where they performed virtual screening on a library of ∼3000 FDA-approved drugs searching for molecules that exhibit the property of being able to target SARS-CoV-2 G4-forming RNA. This is a four-stranded structure in the coronavirus protein that is critical for its function, rendering it a valid pharmaceutical target. This approach is considered ligand-based because the researchers disregarded the actual structure of the target but instead sought to find drugs with similar structural features to known active RNA G4 ligands using pharmacophore modeling. This process resulted in 15 putative RNA G4 drugs, of which 5 had already been evaluated as possible anti-SARS-Cov-2 agents.

In this paper, ligand-based drug repurposing was likewise conducted for the psychiatric disease obsessive-compulsive disorder (OCD). For this study, the Python library DeepPurpose [4] was utilized. DeepPurpose is a deep-learning toolkit which allows the facilitation of drug-target interaction (DTI) prediction tasks in a comprehensive manner from start to finish. Other state-of-the-art deep-learning frameworks used to perform drug discovery-related tasks include DeepChem [5] and Torchdrug [6]. More specifically, graph neural network methodologies were used to represent a molecule bound to an OCD-relevant target comprised of four features in order to be used as input for a neural network. In this manner, three models were created that were able to predict the binding affinity of a molecule to the targets used in the training phase. Then, an ensemble of these models was created and externally validated using random sampling. Finally, case studies of molecules with high scores were bibliographically investigated and were found to be in several forms linked to OCD.

## 2. Materials

### 2.1. OCD-related targets used

OCD is a psychiatric disease in which individuals experience intrusive thoughts compelling them to perform certain rituals in order to temporarily relieve the stress caused by it. This syndrome detrimentally affects afflicted patients as the rituals it induces, which often last one hour or more, significantly diminish their quality of life. It is estimated that 2.3% of people are affected by OCD in their lives [7] and given its debilitating nature, pharmacological treatment is of paramount importance.

The first line of defense against OCD are drugs targeting the serotonin transporter (SERT) such as selective serotonin reuptake inhibitors (SSRIs). Some of them are sertraline, fluoxetine, fluvoxamine, and paroxetine [8]. However, there is a portion of patients who exhibit limited or even no response to these drugs. To overcome this resistance, atypical antipsychotic drugs targeting the dopamine D2 receptors can be used as a form of augmentation therapy to the typical prescription of serotonin inhibitors. One example is olanzapine, a drug originally used to treat the symptoms of schizophrenia, which has been shown to improve symptoms of OCD [9], thus constituting D2 as a potential pharmacological target for the disease. Furthermore, Fluvoxamine, which is, as mentioned above, among the most common drugs used to treat OCD, has also been shown to target the D2 receptors in addition to its primary activity against SERT [10].

Lastly, there is evidence that drugs which modulate the glutamate receptor N-methyl-D-aspartate (NMDA) may also possess therapeutic utility for treatment-resistant forms of OCD such as memantine (which is used against Alzheimer's disease), riluzole (which is used against amyotrophic lateral sclerosis), and ketamine (which is an anesthetic but also an antidepressant and analgesic, depending on the dosage) [8]. Therefore, the targets that were used are SERT, D2, and NMDA, and one model per target was constructed.

### 2.2. Data used for training

Drug activity data against SERT, D2, and NMDA were obtained from the public database BindingDB [11], which contains information about over 2.8 million small molecule-protein experimental interactions. In the creation of a classification machine learning model, positive training set data holds significance but the quality of negative training items is equally crucial. Given the fact that drugs can impact various aspects of the body, selecting random drugs as negatives does not ensure the avoidance of interaction with the targets engaged by active items and, for this reason, the public database DUD-E [12] was employed. This database contains a curated list of “decoys” for 102 targets—molecules which are guaranteed to have no interaction with the target in question, making them optimal candidates for negative items in this study. All molecules used were in the SMILES format [13], a chemical notation representing the structure of a molecule with ASCII characters. For example, the SMILES representation of aspirin is CC(=O)OC1=CC=CC=C1C(=O)O.

As mentioned before, for each target, a separate model was constructed. For the SERT and D2 models, 200 active items were selected from BindingDB with a binding activity of Ki ≤ 10 nM and 200 inactive items from DUD-E. For the NMDA model, 152 active items were selected from BindingDB with a binding affinity of Ki ≤ 12 nM and 150 inactive items from DUD-E. Ki is the inhibitory constant, which is a measure of how potently a substance inhibits a biochemical function with lower values denoting stronger interaction. According to a number of studies, Ki values in the nanomolar range, similar to the items in the training dataset, denote strong binding affinity [14].

Lastly, the items selected as active for the external validation of the ensemble model were all drugs classified as “SERT inhibitor”, “D2 inhibitor”, or “NMDA inhibitor” in the database DrugBank (Wishart et al., 2018). The number of drugs in those categories was 109, 65, and 18, respectively, resulting in 154 unique drugs in total. The items selected as non-active were 5744 drugs from the database Drug Repurposing Hub (Corsello et al., 2017), which were not annotated as neurological or psychiatric. It should be noted that none of the drugs used for the external validation were used in the training dataset.

## 3. Methods

### 3.1. SERT, D2, and NMDA models

The first step of the pipeline of this study was to represent the input molecules, which were in SMILES format, into a list containing 4 features, one atom-level feature, one bond-level feature, the neighbor information of every atom, and the neighbor information of every bond. DeepPurpose offers an extensive selection of molecular encoding methods, with some of them being a MPNN [15], which stands for a message-passing neural network, which is a neural network architecture specifically tailored for making predictions on graph-structured data by iteratively passing and updating information between connected nodes in the graph (called “neighbors”), the convolutional neural network on SMILES, and Morgan fingerprints. The MPNN encoding was selected because it showed one of the most consistent performances compared to all of the available encodings. The data was split into three groups: train, validation, and test groups in percentages of 70%, 10%, and 20%, respectively.

**Figure 1.**
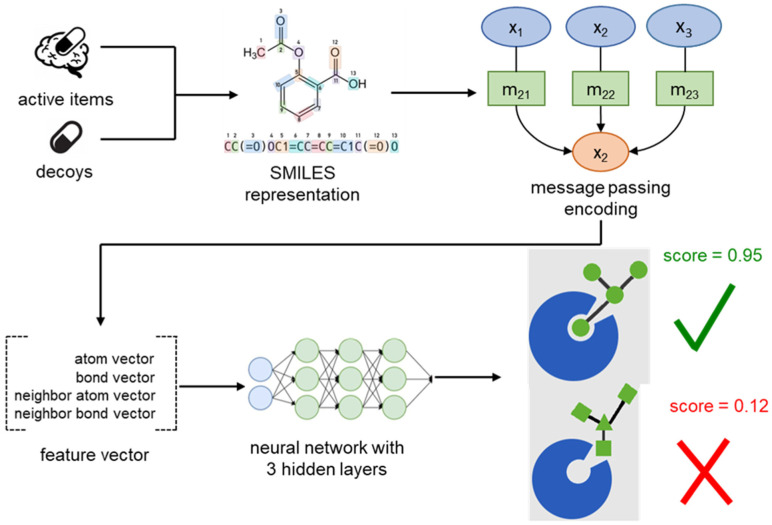
The workflow of the model from dataset creation to finish.

Then, this encoding was fed into a neural network with 3 hidden layers with sizes of 1024, 1024, and 512 nodes, respectively. The number of training epochs was 100, the learning rate was set to 0.001, and the batch size was 128 with the loss function being the mean squared error (MSE). The output was a real number between 0 and 1 denoting how strong the input molecule is predicted to bind with the target in question. The threshold used to differentiate positive and negative predictions was 0.5. This process, depicted in Figure 1, was conducted three times to construct three models in total, one using SERT as a target, one using D2, and one using NMDA.

### 3.2. Ensemble model

After the creation of the individual models, an ensemble model was created with the following formula (for each input molecule):

Ensemble score=(Score(SERT)+Score(D2)+Score(NMDA))/3.

For its evaluation, multiple threshold points for the limit of what constituted a positive or negative item were considered. Because negative items vastly outnumbered positive ones in the ensemble validation set, multiple runs were conducted. In each repetition, 154 items were randomly selected out of the 5744 total negative items to match the 154 positives in order to construct a balanced set. This process was performed 1000 times and the metrics obtained were the average values of each run.

## 4. Results

### 4.1. Individual model performance metrics

After each model was trained for 100 epochs, it underwent testing on the samples that were reserved during the splitting phase. In Table 1, the associated performance metrics are displayed, which are the AUROC (area under the receiving operating characteristic), AUPRC (area under the precision-recall curve), and F1 (the harmonic mean of the precision and recall scores).

**Table 1.** Performance metrics of the models created.

	AUROC	AUPRC	F1
SERT model	0.96	0.96	0.96
D2 model	0.97	0.96	0.92
NMDA model	0.97	0.95	0.91

### 4.2. External validation of the ensemble model

Τhe results of the external validation of the ensemble model after 1000 repetitions for various threshold values that indicated a positive result are displayed on Table 2, as described in section 3.2. More specifically, the labels TP, FN, TN, and FP stand for the average of true positives, false negatives, true negatives, and false positives after 1000 repetitions choosing 154 random molecules that are not for psychiatric or neurologic use out of the total 5744. Likewise, the labels Accuracy, Precision, Sensitivity, Specificity, and F1 stand for the average of those metrics after 1000 repetitions. It was observed that, as the number of repetitions increased, those values exhibited a tendency to converge to a specific number without displaying any signs of divergence.

**Table 2.** Performance metrics of the ensemble model.

Threshold	TP	FN	TN	FP	Accuracy	Precision	Sensitivity	Specificity	F1
0.5	134	20	11.6	8.3	0.83	0.94	0.87	0.58	0.9
0.6	129	25	16	8.9	0.81	0.93	0.83	0.64	0.88
0.7	125	29	21.5	7.4	0.8	0.94	0.81	0.74	0.87
0.8	108	46	36	9	0.72	0.92	0.7	0.8	0.79
0.9	91	63	53.9	9	0.66	0.9	0.59	0.85	0.71
0.95	75	79	67.2	7.7	0.62	0.9	0.48	0.89	0.63

## 5. Discussion and conclusions

One of the biggest challenges in the treatment of OCD is the non-responsiveness that some patients exhibit to the medications typically prescribed. While SERT remains a central OCD drug target, refractory cases prompt the consideration of alternative pathways such as D2 and NMDA. This phenomenon justifies the creation of the ensemble model in this study as it offers a comprehensive evaluation of potential OCD drug candidates by aggregating scores from models targeting not only one but three key pathways. This way, the ensemble model provides a nuanced perspective by identifying drugs with high scores across multiple targets, suggesting a potential for broader efficacy in treating OCD.

The individual models presented a sufficient performance with all three of them having an F1 score above 0.9. This suggests that they concurrently attained high precision and recall (sensitivity). Regarding the evaluation of the ensemble model, multiple threshold values differentiating positive from negative predictions were considered. In the context of the objective in this study, a greater tolerance was exhibited toward Type II errors, wherein the predicted class was negative while the actual class was positive. The minimization of those errors is particularly warranted in scenarios such as cancer detection, where it is imperative to ensure a high degree of confidence in affirming the true negativity of a prediction. In tasks such as drug-target prediction, the focus is shifted to the Type I errors to ensure the highest level of certainty that a predicted active status for a drug truly corresponds to its genuine activity, underscoring the importance of reducing false positives in the present scenario under consideration. It was noted that the different threshold values examined did not meaningfully affect the number of false positives predicted by the ensemble model, which were the most important values to minimize. As the threshold number increased, there was a notable reduction in the occurrences of false negatives, which were not particularly important in this case, accompanied by a rapid decline in the number of true positives. Therefore, users may opt to err on the side of a lower threshold in order to obtain a larger pool of positive predictions to be used for subsequent analysis.

Using the ensemble model to screen 6111 drugs from the Drug Repurposing Hub, mentioned in section 2.2, revealed that a portion of the compounds that scored the highest were also linked according to bibliography to OCD in some form or function. Indicatively, the drug pitolisant displayed an ensemble score of 0.9987. Pitolisant was originally identified as a histamine-3 receptor (H3R) competitive antagonist and was successfully used against narcolepsy [16]. A recent study, though, suggested that it displays effectiveness against Prader-Willi syndrome, which, among other symptoms, causes obsessive-compulsive behavior [17]. Compulsion to overeat and obsessions of insatiable hunger are almost always present, requiring the locking of nearby refrigerators, pantries, and food cabinets by patient guardians essential for the management of the syndrome [18].

Blonanserin is an atypical antipsychotic drug developed in Japan and approved by the PMDA in 2008 [19]. It is a confirmed SERT and D2 receptor antagonist, as indicated also by its ensemble model score of 0.9997. Blonanserin was found to be an effective treatment for somatic symptom disorder (SSD) [20], which is a disorder closely related to OCD causing patients to be obsessed with physical symptoms such as shortness of breath, fatigue, and pain, often resulting in overbearing stress and difficulty in executing daily functions.

Alverine, originally a gastrointestinal antispasmodic drug, displayed an ensemble score of 0.9982. In a network-based drug-repurposing study [21], Alverine was experimentally validated to have antidepressant-like effects in mouse models of depression and was also confirmed to be a SERT interactor, validating the prediction of the SERT model which had a score of 0.999.

Biperiden is an antiparkinsonian drug that improves muscle control and reduces stiffness caused by the disease [22]. Biperiden displayed an ensemble score of 0.998 and was observed to successfully alleviate the acute akathisia, the inability to stand still, which is caused as a side effect by fluvoxamine [23]. Fluvoxamine is one of the main drugs used to treat OCD, and therefore the demonstrated ability of biperiden to decrease its side effects constitutes it as a valid OCD drug candidate, albeit because of indirect reasons. An even more effective drug in reducing the side effects of fluvoxamine is mianserin, which displayed an ensemble score of 0.996. Mianserin was not only successful in treating the persistent form akathisia (which is more severe than the acute form mentioned above) caused by fluvoxamine [23] but, according to a drug trial [24], it outperformed clomipramine, which is a typical drug prescribed against OCD.

The above bibliographic validation of selected case studies concludes this study. The utility of the work presented lies in the fact that the use of the ensemble model can substantially decrease the number of drugs that have to be considered in a virtual screening project by filtering out items with low scores. For instance, out of 6111 drugs in the Drug Repurposing Hub database mentioned before, only 3489 display an ensemble score above 0.5 Therefore, its application filters out more than 40% of the initial candidates, potentially reducing the cost and time commitments involved. Moreover, the individual models demonstrate commendable performance metrics and can find applicability in various diseases where relevant targets are present, including OCD. Some further steps that could be taken would be to consider using other deep-learning frameworks such those mentioned in the introduction, using the model to test nutraceuticals, natural compounds, and other compounds, as well as testing the model against other OCD-relevant targets, too. Finally, it is important to note that laboratory validation, whether in vitro or in vivo, remains imperative for a comprehensive assessment of the viability of the selected candidates.
